# Estimating the Timing of Early Simian-Human Immunodeficiency Virus Infections: a Comparison between Poisson Fitter and BEAST

**DOI:** 10.1128/mBio.00324-20

**Published:** 2020-03-24

**Authors:** Elena E. Giorgi, Hui Li, Tanmoy Bhattacharya, George M. Shaw, Bette Korber

**Affiliations:** a Los Alamos National Laboratory, Los Alamos, New Mexico, USA; b New Mexico Consortium, Los Alamos, New Mexico, USA; c Perelman School of Medicine, University of Pennsylvania, Philadelphia, Pennsylvania, USA; Johns Hopkins Bloomberg School of Public Health

**Keywords:** evolution, HIV, SHIV, transmission

## Abstract

Many HIV prevention strategies are currently under consideration where it is highly informative to know the study participants’ times of infection. These can be estimated using viral sequence data sampled early in infection. However, there are several scenarios that, if not addressed, can skew timing estimates. These include multiple transmitted/founder (TF) viruses, APOBEC (apolipoprotein B mRNA editing enzyme, catalytic polypeptide-like)-mediated mutational enrichment, and recombination. Here, we suggest a pipeline to identify these problems and resolve the biases that they introduce. We then compare two modeling strategies to obtain timing estimates from sequence data. The first, Poisson Fitter (PF), is based on a Poisson model of random accumulation of mutations relative to the TF virus (or viruses) that established the infection. The second uses a coalescence-based phylogenetic strategy as implemented in BEAST. The comparison is based on timing predictions using plasma viral RNA (cDNA) sequence data from 28 simian-human immunodeficiency virus (SHIV)-infected animals for which the exact day of infection is known. In this particular setting, based on nucleotide sequences from samples obtained in early infection, the Poisson method yielded more accurate, more precise, and unbiased estimates for the time of infection than did the explored implementations of BEAST.

## Abstract (precis)

The inference of the time of infection is a critical parameter in testing the efficacy of clinical interventions in protecting against HIV-1 infection. For example, in clinical trials evaluating the efficacy of passively delivered antibodies (Abs) for preventing infections, accurate time of infection data are essential for discerning levels of the Abs required to confer protection, given the natural Ab decay rate in the human body. In such trials, genetic sequences from early in the infection are regularly sampled from study participants, generally prior to immune selection, when the viral population is still expanding and genetic diversity is low. In this particular setting of early viral growth, the Poisson method is superior to the alternative approach based on coalescent methods. This approach can also be applied in human vaccine trials, where accurate estimates of infection times help ascertain if vaccine-elicited immune protection wanes over time.

## INTRODUCTION

Estimates of within-host infection times can be highly relevant to HIV-1 clinical trials. For example, in testing protection mediated through passively administered antibodies (Abs), which have a natural decay rate in the human body, correctly inferring the time since infection is critical to determining Ab levels that are required for protection. There are currently different strategies available to estimate the time since infection. The BEAST software by A. J. Drummond and A. Rambaut (1) is widely used to estimate the evolutionary rate and molecular clock for sets of genetic sequences. BEAST implementations of either the coalescent skyline model (2) or the birth-death skyline model (3) have been used to reconstruct the dynamics of the epidemic spread at the population level of rapidly evolving viral infections (1, 4). Alternatively, given the stringent genetic bottleneck that HIV-1 encounters at transmission (5), the virus’s mutation rate, and its subsequent rapid evolution, the timing of the most recent common ancestor (MRCA) in a phylogenetic tree can be also used as the basis of a reasonable strategy to estimate an individual’s time of infection (6).

We have developed a simple strategy to estimate time of infection based on early viral sequence diversity and a fixed mutation rate (5, 7). Efficacy trials where the main outcome is protection from HIV-1 infection offer a unique setting where study participants are sampled frequently and, if infection occurs, viral sequences from the host can be obtained within a narrow time window early in infection. Such samples are often obtained prior to the onset of the early adaptive immune responses, which is important because once deployed, such immune responses impose strong and dynamic host-specific selective pressure that biases assumptions of a molecular clock (8, 9). In that time period, within 1 to 2 months of infection, viral diversity is too low to appropriately inform coalescence-based methods. In this scenario, when the viral population is expanding prior to the onset of immune pressure, as an alternative to the existing approaches based on coalescent methods, one can assume a Poisson process of random accumulation of mutations relative to transmitter/founder (TF) viruses (5). This is the basic assumption of our previously described Poisson Fitter (PF) tool (5, 7), which, in the setting of very early infection, performs at its best for estimating the time from infection.

Here, we illustrate the application of a pipeline that incorporates PF timing estimates and addresses three additional issues that can confound phylogeny-based methods. These include the following: within-host recombination, hypermutation, and infections established by multiple TF viruses. Both recombination and APOBEC (apolipoprotein B mRNA editing enzyme, catalytic polypeptide-like)-mediated G→A mutations are common in HIV and can artificially skew tree-branch lengths (10). APOBEC-mediated mutations have distinctively high mutation rates (11). They often manifest as severely hypermutated sequences (12), which can be readily identified and easily removed from an alignment. A less-well-known manifestation of APOBEC-mediated hypermutation is a more subtle enrichment of G→A mutations across an entire alignment from a particular time point (5). In such cases, sequences may be perfectly viable, with only a few G→A substitutions in a given sequence. However, the mutational pattern across the full sample is dominated by G→A substitutions enriched only in the context of APOBEC motifs, inappropriately inflating the overall mutation rate within that sample (5).

Our PF pipeline includes screening for within-host recombination when multiple TFs are present, as well as both of the manifestations of APOBEC enrichment just described. This screening is performed by the use of the LANL tools RAPR (10) and Hypermut (13). In addition, we accommodate situations where multiple TFs establish an infection, in which case each distinct lineage is considered to represent a separate Poisson process (5, 14).

We illustrate our methods using simian-human immunodeficiency virus (SHIV) sequences from 28 recently infected rhesus macaques (RMs) for which the exact inoculation day was known and compare the accuracy of the PF and coalescent strategies for estimating the number of days since infection for each animal. We show that both PF and BEAST estimates of the day of infection are improved by prescreening and filtering out both kinds of APOBEC G→A mutational enrichment and by separating distinct TF lineages. In the latter case, the PF pipeline method, but not the BEAST method, incorporates steps to detect and accommodate recombination between lineages through the use of the RAPR tool (10). In our analyses, we explored different BEAST parameters and priors, following strategies published in recent studies with outbreak data sets analogous to ours (3, 4). While other yet-unexplored strategies may yield different results, in our explorations we found that in all instances PF performed more accurately and efficiently than did BEAST in estimating days since infection based on sequence diversity sampled very early in infection.

## RESULTS

**FIG 1:**
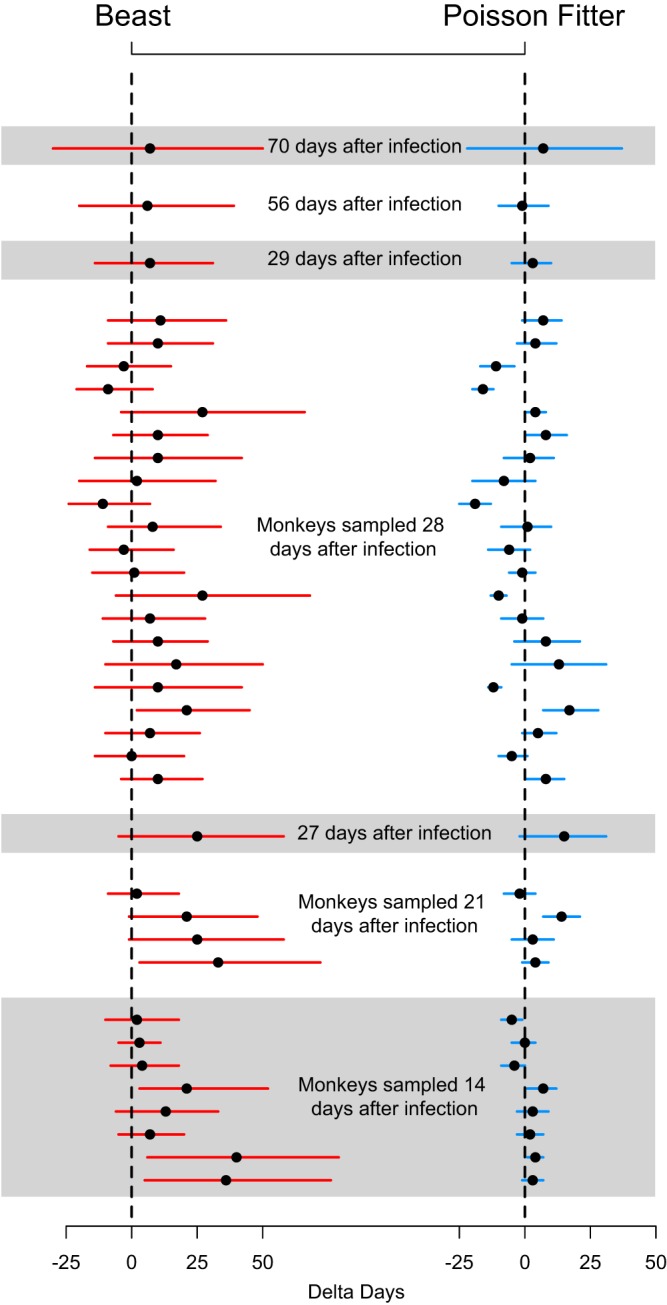
Comparison between PF and BEAST estimates. Infection time estimates and 95% CIs are shown for each animal sample. PF estimates are shown in blue and BEAST estimates in red. Samples are ordered by time since inoculation, with the most recent samples (2 weeks) at the bottom and the oldest (10 weeks) at the top. For example, time estimates for 8 monkeys sampled precisely 14 days after infection are shown in the bottom gray rectangle in the figure, time estimates for 4 monkeys sampled at 21 days after infection are shown in the rectangle above, and so on. Vertical dashed lines indicate the inoculation times and black dots the estimated time (to the left of the dashed line for estimated durations that are shorter than the actual time and to the right for estimated durations that are longer than the actual time). Black dots that appear on the vertical dashed line indicate time estimates that coincide with the actual time of infection. The width of the red and blue lines represents the width of the 95% BEAST credible intervals and 95% PF confidence intervals, respectively.

**FIG 2:**
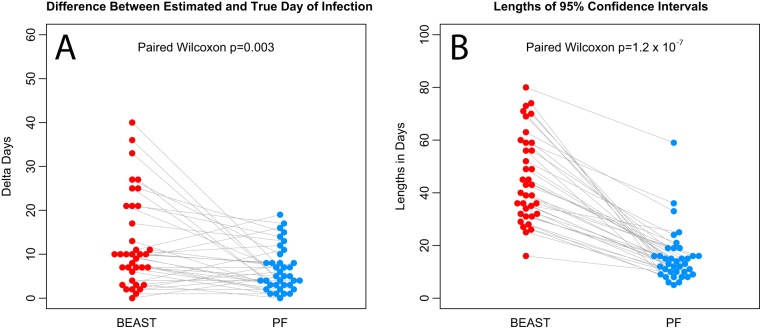
Differences in PF and BEAST estimates. (A) Plots of differences between the estimated times and the true inoculation time in BEAST (red) and PF (blue). BEAST differences tend to be higher (*P* = 0.003 by paired Wilcoxon test). (B) Lengths of BEAST 95% credible intervals (red) and PF 95% confidence intervals (blue). BEAST credible intervals tend to be longer (*P* = 1.2 × 10^−7^ by paired Wilcoxon test).

Twenty-eight RMs were infected either intravenously or intrarectally with SHIVs (15) and were serially sampled at different time points, for a total of 51 sequence sets sampled between 2 and 12 weeks following inoculation (Tables 1 and 2). For our PF-BEAST timing estimate comparisons (Fig. 1 and 2), we focused on samples with mutational events conforming to a Poisson distribution, so that the accumulation of observed mutations was consistent with random mutational events prior to host-specific immune selection. There were 28 Env sequence sets sampled from the first available time point from each animal. All of these were sampled within 4 weeks from infection, and only one of these first time point sets did not fit a Poisson distribution after controlling for APOBEC mutations and multiple infections. Twenty-three later time points were also sampled, and from these, 10 additional sequence sets were found to also conform to a Poisson model. This yielded a total of 37 samples, including a total of 1,437 Env sequences, all generated by single-genome sequencing (5). We analyzed each sample in PF, assuming a fixed mutation rate of 2.16 × 10^−5^ per site per generation (5), which was calculated from the *in vitro* estimate obtained by Mansky and Temin (16) after excluding insertions and deletions. In our experience, we have found this rate to yield accurate timing estimates, despite its having been derived *in vitro* and averaged across transitions and transversion. When found to improve the fit to the Poisson model, new alignments with positions in the APOBEC context removed were used for both the PF and BEAST runs. Lineages arising from multiple founder viruses were each considered separately. While we tested different settings in BEAST 2.6.0 (see Materials and Methods), the strategy that yielded the best results was use of the GTR + Γ + I substitution model, with a strict clock, with the substitution rate fixed to 2.16 × 10^−5^ per site per generation (one HIV generation = ∼1.5 days) and a contemporary birth-death skyline prior, as previously described (3).

**TABLE 1:** Comparison of estimated days from infection obtained through Poisson Fitter and BEAST^*a*^

Animal ID	Inoculum	No. of wks	Nseq	Infection date	No. of days	No. of PF days (95% CI)	GOF *P* value	No. of BEAST days (95% CI)
RM08N021	SHIVBG505, Ref	4	50	19 May 2016	28	36 (28, 43)	0.94	38 (24, 55)
RM10N011	SHIVBG505, Ref	4	54	19 May 2016	28	23 (18, 29)	0.51	28 (14, 48)
RM131	SHIV191859, Mix	2	35	NA	14	17 (13, 21)	0.426	50 (19, 90)
RM131	SHIV191859, Mix	3	23	NA	21	25 (20, 30)	0.923	54 (24, 93)
RM138	SHIV191859, Mix	2	38	NA	14	18 (15, 21)	0.955	54 (20, 92)
RM138	SHIV191859, Mix	3	30	NA	21	24 (16, 32)	0.74	46 (20, 79)
RM194	SHIV191859, Ref	4	52	NA	28	33 (27, 40)	0.08	35 (18, 54)
RM196	SHIV191859, Ref	4	41	NA	28	45 (35, 56)	0.92	49 (30, 73)
RM196	SHIV191859, Ref	10	12	NA	70	77 (48, 107)	0.08	77 (40, 120)
RM41216	SHIV40100, Mix	4	37	27 June 2016	29	32 (24, 39)	0.75	36 (15, 60)
RM43335	SHIVBG505, Mix	4	31	20 October 2015	28	16 (14, 19)	0.563	38 (14, 70)
RM5694	SHIVCH505, Ref	2	71	17 May 2017	14	16 (11, 21)	0.14	21 (9, 34)
RM5694	SHIVCH505, Ref	3	71	17 May 2017	21	35 (28, 42)	0.27	42 (20, 69)
RM6072	SHIVCH505, Mix	4	22	24 February 2015	27	42 (25, 58)	0.8	52 (22, 85)
RM6434	SHIVBG505, Mix	4	36	20 October 2015	28	41 (23, 59)	2.E−16	45 (18, 78)
RM6442	SHIVBG505, Mix	4	24	20 October 2015	28	36 (24, 49)	0.09	38 (21, 57)
RM6446	SHIVBG505, Mix	4	30	20 October 2015	28	30 (21, 38)	0.47	35 (17, 56)
RM6454	SHIVBG505, Mix	4	26	20 October 2015	28	18 (15, 21)	0.965	55 (22, 96)
RM6706	SHIVBG505, Ref	4	46	28 April 2016	28	27 (22, 32)	0.34	29 (13, 48)
RM6708	SHIVBG505, Ref	4	43	28 April 2016	28	22 (14, 30)	0.09	25 (12, 44)
RM6715	SHIVBG505, Ref	4	34	28 April 2016	28	29 (19, 38)	0.82	36 (19, 62)
RM6717	SHIVBG505, Ref	4	22	19 May 2016	28	9 (3, 15)	0.87	17 (4, 35)
RM6718	SHIVBG505, Ref	4	22	28 April 2016	28	20 (8, 32)	0.045	30 (8, 60)
RM6719	SHIVBG505, Ref	4	29	28 April 2016	28	30 (20, 39)	0.21	38 (14, 70)
RM943	SHIVCH848, Mix	2	34	25 April 2018	14	17 (11, 23)	0.86	27 (8, 50)
RM943	SHIVCH848, Mix	4	36	25 April 2018	28	36 (28, 44)	0.85	38 (21, 57)
RM944	SHIVCH848, Mix	2	42	25 April 2018	14	21 (15, 26)	0.96	35 (17, 66)
RM944	SHIVCH848, Mix	4	40	25 April 2018	28	32 (28, 36)	0.148	55 (24, 94)
RM945	SHIVCH848, Mix	2	42	25 April 2018	14	10 (5, 14)	0.83	18 (6, 32)
RM945	SHIVCH848, Mix	4	48	25 April 2018	28	12 (8, 16)	0.89	19 (7, 36)
RMT283	SHIVBG505, Ref	4	29	28 April 2016	28	17 (11, 24)	0.77	25 (11, 43)
RMT775	SHIVCH505, Ref	2	75	17 May 2017	14	14 (9, 18)	0.85	17 (9, 25)
RMT775	SHIVCH505, Ref	3	51	17 May 2017	21	19 (13, 25)	0.4	23 (12, 39)
RMT775	SHIVCH505, Ref	8	51	17 May 2017	56	55 (46, 65)	0.55	62 (36, 95)
T682	SHIV1086, Mix	2	32	9 September 2016	14	9 (5, 13)	0.55	16 (4, 32)
T929	SHIV1086, Ref	4	38	1 October 2017	28	32 (25, 40)	0.85	38 (19, 59)
T930	SHIV1086, Ref	4	40	1 October 2017	28	35 (27, 42)	0.99	39 (19, 64)

a For 3 RMs, the infection date was not available (NA), although the days following infection were known. ID, identifier; Nseq, number of sequences; Mix, mixture; Ref, reference; GOF, goodness of fit; CI, confidence intervals for PF, credible intervals for BEAST.

**TABLE 2:** Inoculation schedule and inoculum type for 28 animals^*a*^

Animal ID	Inoculum		Stock type	Dose		Route
	SHIV strain	Position 375 variant(s)		ng of p27 used for each variant	Total ng of p27 used for each animal	
RM196	SHIV 191859	S and M	293T	250	1,000	i.r.
	SHIV 191859 gp41	S and M				
RM194	SHIV 191859	S and M	293T	250	2,000	i.r.
	SHIV 191859 gp41	S and M				

RM131	SHIV 191859	S, M, H, Y, F, and W	293T	166	1,000	i.v.
RM138	SHIV 191859	S, M, H, Y, F, and W	293T	166	1,000	i.v.
RM6434	SHIV BG505 332N	S, M, H, Y, F, and W	293T	50	300	i.v.
RM6442	SHIV BG505 332N	S, M, H, Y, F, and W	293T	50	300	i.v.
RM6446	SHIV BG505 332T	S, M, H, Y, F, and W	293T	50	300	i.v.
RM6454	SHIV BG505 332N	S, M, H, Y, F, and W	293T	50	600	i.v.
	SHIV BG505 332T	S, M, H, Y, F, and W				
RM43335	SHIV BG505 332N	S, M, H, Y, F, and W	293T	50	600	i.v.
	SHIV BG505 332T	S, M, H, Y, F, and W				
RM6718	SHIV BG505 332N	Y	RhCD4^+^ T cells	8	8	i.r.
RM6719	SHIV BG505 332N	Y	RhCD4^+^ T cells	8	8	i.r.
RMT283	SHIV BG505 332N	Y	RhCD4^+^ T cells	1.5	1.5	i.r.
RM6715	SHIV BG505 332N	Y	RhCD4^+^ T cells	8	8	i.r.
RM6706	SHIV BG505 332N	Y	RhCD4^+^ T cells	1.5	1.5	i.r.
RM6708	SHIV BG505 332N	Y	RhCD4^+^ T cells	1.5	1.5	i.r.
RM6717	SHIV BG505 332N	Y	RhCD4^+^ T cells	8	8	i.r.
RM08N021	SHIV BG505 332N	Y	RhCD4^+^ T cells	1	1	i.r.
RM10N011	SHIV BG505 332N	Y	RhCD4^+^ T cells	1	1	i.r.

RM6072	SHIV CH505	S, M, H, Y, F, and W	293T	500	3,000	i.v.
RM5694	SHIV CH505	H in 3 different backbones	293T	5	15	i.v.
RMT775	SHIV CH505	H in 3 different backbones	293T	5	15	i.v.

RMT943	SHIV CH848 1017	H	293T	50	100	i.v.
	SHIV CH848 1017DT	H				
RMT944	SHIV CH848 1017	H	293T	50	100	i.v.
	SHIV CH848 1017DT	H				
RMT945	SHIV CH848 1017	H	293T	50	100	i.v.
	SHIV CH848 1017DT	H				

RMT682	SHIV 1086	S, M, H, Y, F, and W	293T	50	300	i.v.
RMT929	SHIV 1086	W	293T	50	50	i.v.
RMT930	SHIV 1086	W	293T	50	50	i.v.

RM41216	SHIV 40100	S, M, H, Y, F, and W	293T	50	300	i.v.

a The inocula for RM5694 and RMT775 were identical in *env* gp160 sequence. i.r., intrarectal; i.v., intravenous.

**FIG 3:**
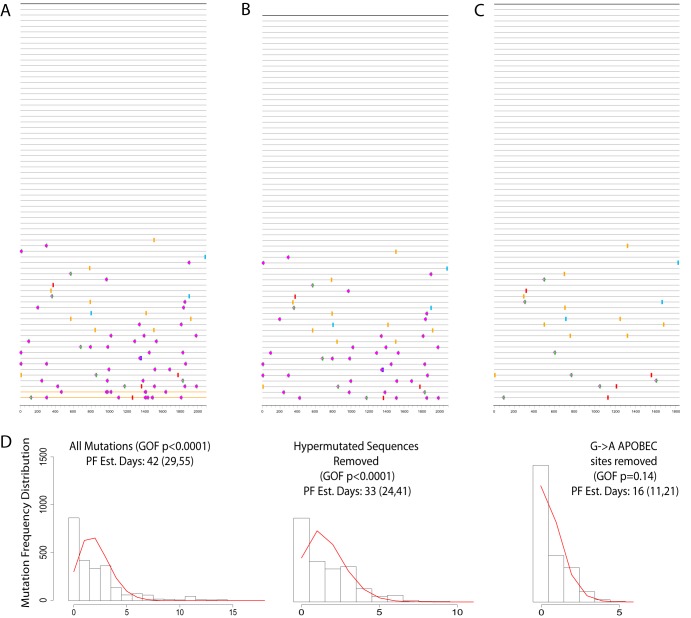
G→A mutations in day 14 sample from animal RM5694. Top panels show highlighter plots of the sequences taken 14 days after infection compared to the inferred TF viruses. Each sequence is represented by a horizontal segment, and mutations from the TF (top segment, in black) are shown as colored tic marks. (A) Highlighter plot of all sequences in the sample. G→A mutations are shown by green tic marks, and they are additionally marked by a pink circle when they are in the APOBEC context or by an open diamond when they are not. Pink circles are overwhelmingly more numerous than open diamonds (totaling only 7), a clear indication that G→A APOBEC mutations were happening at a significantly higher rate than all other mutations. (B) Highlighter plot after removal of the two hypermutated sequences (shown in orange at the bottom of panel A). Despite the hypermutated sequences having been removed, the sample still presents a high number of APOBEC mutations, explaining why a Poisson distribution still does not fit well. (C) Highlighter plot of the sample after removal of all positions in the APOBEC context, in other words, in all columns in the alignment where the TF carries a guanine followed by either a thymine or an adenine. (D) Frequency distributions of the mutation counts for each of the representations of the sample described above, represented as follows: left, all sequences; middle, all but the two hypermutated sequences; right, all sequences with the APOBEC G→A positions removed. A good fit with a Poisson distribution and an accurate infection time estimate were achieved only after the columns representing the potential for APOBEC-mediated mutations were removed, as indicated by the GOF *P* value of 0.14.

We found 16 of 37 (43%) RM samples to be significantly enriched for APOBEC mutations (Fig. 3). This was a higher proportion than we had previously observed in humans (5). For all 16 samples, a good Poisson fit was restored after removal of columns from the alignment that contained G’s in the transmitted/founder virus that were embedded in a motif that enables APOBEC-mediated hypermutation. For two additional samples, removing positions in an APOBEC motif improved the Poisson fit. Only 1 of the 37 total samples did not yield a good Poisson fit even after screening for APOBEC enrichment. Among the 36 samples that fit a Poisson distribution, the inoculation date for 26 (72%) fell within the 95% confidence interval (CI) of the PF estimated infection time (Table 1). Of the 26 samples that yielded a good estimate, 24 had been taken 2 to 4 weeks from inoculation and the remaining 2 at 8 and 10 weeks, suggesting that earlier samples generally enable more accurate timing estimates (Fig. 1). Of note, among the samples taken later, most deviated from a Poisson distribution, and other than the two aforementioned samples, none taken at week 8 or later fit a Poisson distribution. The deviation from the Poisson distribution in these cases was likely due to the onset of host-specific immune selection.

From the BEAST runs, for 32/37 samples (86%) the inoculation times fell within the 95% CI of the estimated time of infection. However, this increase relative to PF was largely due to the fact that the credible intervals yielded by BEAST were generally wider than PF confidence intervals (Fig. 1 and 2; see also Table 1). In fact, comparing the absolute differences in estimated days since infection and in known inoculation days between PF and BEAST, we found the PF differences to be statistically significantly lower (*P* = 0.003 by paired Wilcoxon test) (Fig. 2), and the PF 95% intervals statistically significantly narrower than the BEAST intervals (*P* = 1.2 × 10^−7^ by paired Wilcoxon test) (Fig. 2). In addition, using the previously described settings, BEAST yielded time estimates biased upward of the time between sampling and the day of infection (31/36 cases, binomial *P* = 1.3 × 10^−5^), while the PF estimates did not present this bias (22/36, binomial *P* = 0.24).

Twelve of the RMs had been inoculated with a mixture of amino acids at position 375 (15), and 3 additional RMs with a mixture of equal amounts of the two closely related SHIV strains that had glycan deletions at positions 133 and 138, for a total of 15 animals inoculated with a mixture instead of a single clone (Table 2). Seven of them retained differences in the corresponding codons at the time of sampling. Under conditions in which multiple TFs establish the infection, BEAST estimates the time since the most recent common ancestor, which, in a natural transmission setting, takes place in the donor, not the recipient. Removing the variable positions in codon 375 from the alignments in our study narrowed the error for the BEAST estimates (Table 3) but did not change the overall conclusions: the results from the paired Wilcoxon tests comparing methods as noted above remained significant (*P* = 0.02 and 1.2 × 10^−7^, respectively), and the data representing the number of days since infection estimated by BEAST were still biased upwards (30/36 samples, binomial *P* = 7 × 10^−5^).

**TABLE 3:** Estimated days since infection and 95% confidence/credible intervals obtained by PF and BEAST, respectively, for animals infected with multiple variants^*a*^

Animal and setting	Estimated no. of days (CI) since infection			
	Site removed		Site not removed	
	Poisson fitter	BEAST	Poisson fitter	BEAST
RM131, no APOBEC, day 14	14 (8, 20)	23 (7, 44)	17 (13, 21)	50 (19, 90)
RM131, day 21	28 (19, 38)	32 (16, 53)	25 (20, 30)	54 (24, 93)
RM138, no APOBEC, day 14	22 (15, 28)	27 (12, 47)	18 (15, 21)	54 (20, 92)
RM6454, day 28	32 (19, 45)	39 (19, 68)	18 (15, 21)	55 (22, 96)
RM944, no APOBEC, day 28	32 (24, 39)	34 (15, 59)	32 (28, 36)	55 (24, 94)
RM43335, day 28	17 (12, 21)	21 (8, 36)	16 (14, 19)	38 (14, 70)
RM6072, no APOBEC, day 28	34 (21, 47)	40 (18, 67)	39 (22, 56)	52 (22, 85)
RM6446, no APOBEC, day 28	27 (19, 35)	32 (15, 54)	30 (21, 38)	35 (17, 56)

a The data in columns 2 and 3 represent results obtained after removing the site(s) at which the distinct inoculum variants differed, whereas the data in columns 4 and 5 represent the results from the original samples, as described in the main text, with the codon at 375 (or, in two cases, at 133 and 138) left in.

## DISCUSSION

We have shown that, in comparisons of infection time estimates yielded by BEAST and PF analyses of early SHIV sequence data for which the exact date of infection was known, the latter was more accurate and precise than the former (Fig. 1). Confidence intervals for PF ranged in length from 5 to 59 days, while no BEAST credible interval was narrower than 24 days and the maximum was 94.5 days. In particular, data from RMs infected with multiple TFs yielded CIs that were among the narrowest in PF (5 to 8 days) and among the widest in BEAST (46.5 to 94.5 days). One reason for this discrepancy is that our Poisson-based methods can be readily extended to multiple TF infections by dividing the alignment data into subsets of separate lineages and then combining the time estimates as previously described (14), while this option is not available in BEAST. Removing the site of diversity across lineages did not resolve this discrepancy between BEAST and PF.

**FIG 4:**
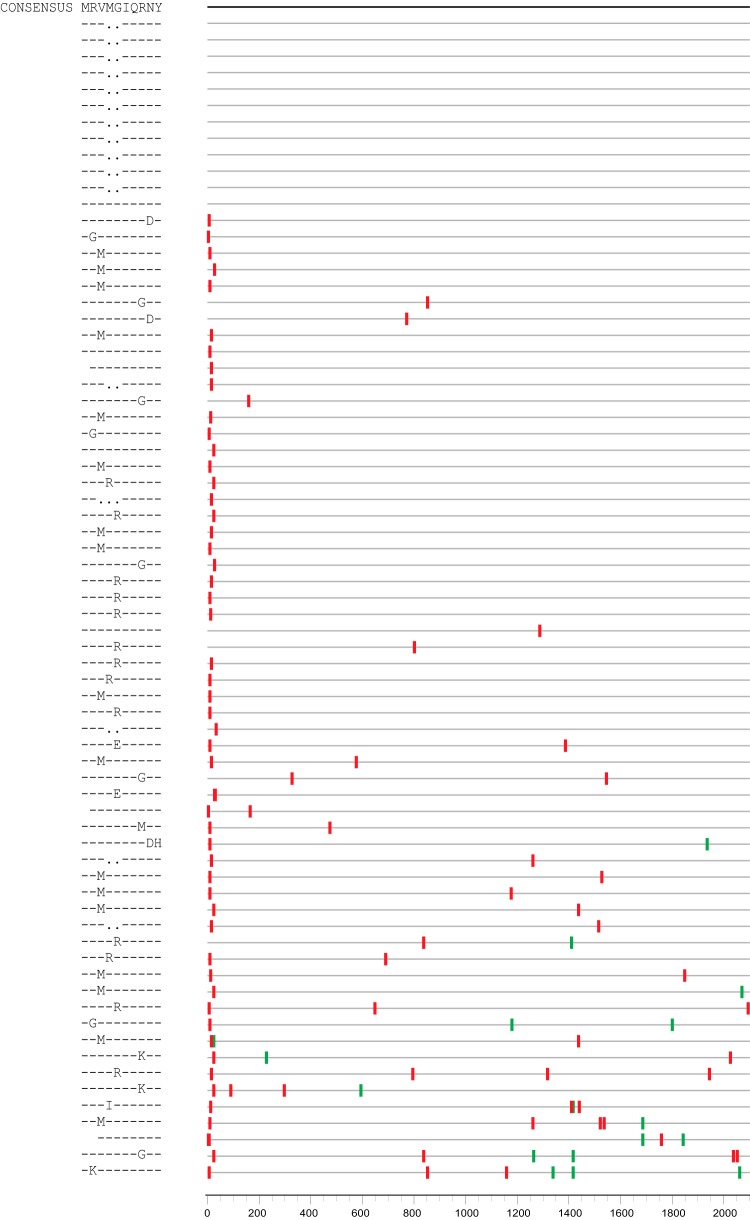
Day 21 synonymous and nonsynonymous mutations from animal RM5694. (Right) Highlighter plot of sequences taken 21 days after the infection, with the putative epitope under immune pressure shown at the very left of the panel, at positions 2 to 10. Each sequence is represented by a gray segment and is compared to the inferred TF (top segment, in black). Red tic marks indicate nonsynonymous mutations and green tic marks synonymous ones. (Left) Mutated amino acids within the putative epitope.

PF performs best with earlier sequence sets, sampled within 3 weeks after infection, which tended to yield the most accurate timing estimates (Fig. 1). As the infection progresses, nonrandom mutational patterns start appearing in response to early selection pressure from the host (Fig. 4). When this happens, the fit of mutational patterns in HIV-1 sequences in early infection may significantly diverge from a Poisson distribution, in which case the goodness-of-fit (GOF) *P* value provided by PF is 0.05 or less. This estimate alerts PF users that the sequence set may be problematic and requires additional attention. Besides immune escapes, there are two additional settings that cause the distribution of sequence distances to diverge from a Poisson distribution, namely, multiple transmitted founders and APOBEC-mediated hypermutation.

The onset of immune escape within the region sequenced can often be identified as a series of highly focused nonsynonymous mutations clustered in a narrow region roughly the size of a linear cytotoxic T-cell epitope (∼10 amino acids; see Fig. 4) (8, 9, 17). Such T cell responses can occur very early in infection, as the peak viremia begins to subside (8, 9). The location of targeted epitopes in the proteome is host specific, depending on both the host HLA and the TF viral sequence, complicating the use of evolutionary models across hosts. In such cases, time-from-infection estimates obtained through the use of the PF tool, because it assumes a fixed mutation rate, would represent overestimates. As an alternative approach, at very early time points in the infection when these responses are localized to a single putative epitope, one can resolve to reestimate the infection time while excluding the region under selection pressure. This would likely restore the Poisson distribution fit; however, the resulting estimate should be presented with a note of caution as it may represent an underestimation due to the bottleneck imposed by the excluded epitope. Additional information gathered from diagnostic markers such as Fiebig staging (18) and the combined strategies described previously by Grebe et al. (19) can help calibrate inferred timing in settings where the original PF assumptions are not completely met. This can be useful in clinical settings, when the early time window preceding the onset of selection may be missed.

APOBEC hypermutation can also result in a violation of the Poisson assumption, and PF provides strategies to restore time estimates by prefiltering sequences (see Fig. 3). APOBEC enrichment can manifest in a single time point alignment as G→A mutations in the APOBEC context scattered throughout many sequences, with those mutations dominating the mutational events across the sample but not necessarily highly enriched in any single sequence. Alternatively, it can take the form of a single hypermutated sequence, which can be readily removed. If present but unaccounted for, either form of APOBEC enrichment can result in overestimates of the time from infection. PF has a built-in way to screen for APOBEC enrichment through the use of the LANL tool Hypermut (13) and, when found, to remove either the enriched sequences or the alignment positions in the APOBEC context. Specifically, in the latter case, for all positions in the alignment where there is a guanine in the consensus/TF sequence within the APOBEC context (namely, positions where the guanine is followed by either an adenine or thymine), the entire column in the alignment is removed. We emphasize that this approach is very different from that of just discounting all observed G→A mutations. Because APOBEC-mediated mutations can happen at a much higher rate than the average across all other mutations as measured by Mansky and Temin (16), APOBEC-mediated G→A mutations cause the Poisson distributions to diverge. In samples with evidence of high APOBEC activity, by removing all columns from the alignment where such mutations might arise, we are excluding the subset of data that is subject to higher mutations rates and are therefore limiting the timing analysis to data for which the baseline average mutation rate of 2.16 × 10^−5^ applies. Since APOBEC acts sporadically on different lineages and at different time points, its effects violate the assumption of independent random mutations. The PF GOF *P* value is a readily accessed indication of the failure of the model, and users are therefore made aware of the presence of a bias and can then determine if this bias is due to APOBEC enrichment using strategies implemented in the PF code. For all of the samples presented here, when removal of APOBEC positions improved the Poisson fit, those positions were removed prior to running BEAST as well for a fair comparison, as represented in Fig. 1 and 2. As a result, BEAST timing estimates were also improved by removing potential APOBEC positions from the alignments (data not shown).

Among 10 samples for which the inoculation time did not fall within the PF-estimated 95% CI of the infection time, 6 estimates were too early and 4 too late. Early onset of positive selection, for example, selection resulting from immune escape driven by the earliest cytotoxic T-cell responses that arise during the course of natural infection (8, 9, 17), can lead to an overestimation of the infection time. However, if immune selection were to happen in an epitope outside the sampled region, a resulting bottleneck could potentially lead to lower diversity in other parts of the genome and therefore to an underestimation of the infection time. One animal in particular, RM5694, which was sampled at 2 and 3 weeks postinfection, provided a good example to illustrate the impact of both hypermutation and immune selection on timing estimates. The 2-week sample originally diverged from a Poisson distribution, yielding a goodness-of-fit (GOF) *P* value of less than 0.0001. Hypermutation screening revealed two APOBEC-enriched sequences (Fig. 3A), both with significantly greater numbers of APOBEC-mediated G→A mutations (*P* = 0.01). However, removing the sequences alone did not resolve the Poisson divergence, as many other G→A mutations in the APOBEC context were present in the rest of the alignment (Fig. 3B). Indeed, in testing for overall hypermutation, we found the sample to be significantly enriched (*P* = 1.3 × 10^−7^). After removal of all APOBEC-mediated G→A mutations, a good fit was restored and an accurate infection time estimate of 16 days was obtained, with a 95% CI of 11 to 21 days and a GOF *P* value of 0.14 (Fig. 3C and D).

Analyzing the data from the next time point for the same animal, sampled at week 3, we observed nonrandom accumulation of mutations at the very beginning of the Env protein, at HXB2 positions 2 to 10 (Fig. 4). In human infections, this has been documented as an HLA-B*0801-restricted cytotoxic T lymphocyte (CTL) epitope (20). While the Poisson distribution was robust with respect to this kind of early divergence and, after once again removing positions in the APOBEC context, yielded a good fit (GOF *P* = 0.27), the resulting time estimate of 35 days (95% CI of 28 to 42 days) substantially exceeded that from the 21-day sample. This could be explained by the fact that the positive selection resulted in more mutations than one would expect after 3 weeks of infection under the assumption of random mutations due to reverse transcriptase error. As discussed above, one possible way to address this issue would be to reanalyze the sample after excluding the region that includes the putative epitope. Such a strategy should be employed with caution, however, as when we applied it, we saw that the time estimate was too low: 16 days, with a CI of 11 to 21 days. While this barely covers the true infection time (21 days), as anticipated, the region outside the epitope had lower diversity than expected at 3 weeks of infection (Fig. 4), likely due to sequences encountering a bottleneck as a consequence of the upstream epitope selection. Had we not had the earlier sample from the day 14 time point for this animal, we would have likely missed the true time of infection. We recommend checking sequences with a visual tool such as Highlighter (5) to visually search for clusters of mutations that are indicative of early epitope responses and, if evident, to treat time estimations from such samples with caution. For further validation of the time estimates, we also recommend combining such information with diagnostic serological data (18, 19) whenever available.

Multiple TFs can also result in a violation of the Poisson assumption. In such cases, phylogenetic trees, paired with Highlighter plots (5), can be used to identify specific lineages. When this happens, each lineage should be treated separately and within-lineage mutations should be counted from each separate TF, as previously described (14). Also of note is that in the presence of very distinctive TFs, recombinants across lineages can cause branch length artifacts in phylogenetic trees. BEAST uses population priors that typically do not incorporate prior knowledge of extremely strong bottlenecks at transmission and do not allow evolution due to recombination. Recombination is very common in HIV and violates the assumptions inherent in most phylogenetic tree reconstructions, potentially confounding infection time estimates. Furthermore, multiple infections by highly related sequences can bias phylogeny-based estimates of the time of transmission also because the lineages would have diverged in the donor in a transmission pair, not the recipient. Importantly, these effects not only are not accounted for using BEAST, they also may go undetected, whereas in PF the combination of a low GOF *P* value with built-in graphics helps the user identify the reasons for the Poisson divergence and identify recombinants and/or multiple founders even when they are highly related. In our present data, while the data from the 7 animals infected with a mixture of inocula (multiple TFs) did not originally yield a good Poisson fit (GOF *P* < 0.05), the fit was restored after considering within-lineage mutations and combining the frequency distributions (see Materials and Methods). In a natural transmission setting, multiple infections with highly similar TFs—such as, for example, ones that differ by only a few bases—may yield overestimates of the time of infection, and PF has built-in diagnostics to alert users to this possibility.

Coalescent models as implemented in BEAST are of great utility in modeling epidemic histories of variable pathogens. However, in the specific setting of attempts to accurately estimate infection times from HIV sequences sampled early in infection, PF was more accurate and precise overall than BEAST (Fig. 1 and 2) based on the early SHIV infection data for which the exact date of infection was known. PF confidence intervals ranged in length from 5 to 59 days, while no BEAST credible interval was narrower than 16 days and were up to 80 days long. In particular, RMs infected with multiple TFs yielded the narrowest CIs (5 to 8 days) in PF and also the widest credible intervals (39 to 74 days) in BEAST. Furthermore, in data sets with few mutations against a large field of highly conserved sequence data, PF performs at its best, and, given the known mutation rate, can even estimate an upper bound on the infection time when no variation is observed. The low precision in BEAST estimates for such data arises partly due to the particular setting, namely, that of very early infections, when viral diversity is too low to inform the coalescent model without strong priors. However, this setting is of particular importance for clinical trials.

In this study, in order to compare BEAST data to PF data, we tried different BEAST priors using the same parameters as those used with PF, as well as additional scenarios where we instead analyzed groups of monkeys together (sampled at the same time and infected with the same inoculum; see Materials and Methods) in order to estimate a common clock rate. While we report here the best BEAST results that we obtained, it is possible that a different choice of model and parameters and settings in BEAST might lead to higher fidelity of the results, so long as the recommended course of preliminary analysis (i.e., elimination of multiple TF lineages, recombinants, and positions in the APOBEC context) is still followed. We point out, however, that the process of choosing ideal parameters and settings is not straightforward. The simplicity of PF, albeit in the limited context of modeling early virus evolution within a host, makes it the tool of choice in this particular setting. Our methods were incorporated in the previously described pipeline (21).

## MATERIALS AND METHODS

The 10 intravenously inoculated animals were described elsewhere (15); 9 additional animals were inoculated using the same protocol (15), and 9 more were inoculated intrarectally with different dosages of SHIV BG505 332N 375Y rhesus macaque CD4 (RhCD4) T-cell-derived stocks (Table 2). Among the RMs, 15 were inoculated with a mixture of amino acids at either position 107 or 375, whereas all other animals were inoculated with identical clones. In addition to all of the first time points included from all animals, later time points were also included in the BEAST/PF comparison if they yielded a good Poisson fit (in other words, if the accumulation of mutations from the original TFs were still random and unbiased by selection), for a total of 37 samples from the 28 animals.

PF was run from the online interface available through the LANL database (https://www.hiv.lanl.gov/content/sequence/POISSON_FITTER/pfitter.html) with a fixed mutation rate of 2.16 × 10^−5^. BEAST version 2.6.0 was used for the comparison, with the following settings: GTR + Γ + I substitution model under a strict clock branch rate model with the clock rate fixed to 2.16 × 10^−5^ substitutions per site per generation (one HIV generation = ∼1.5 days), and contemporary birth-death skyline prior, as described previously (3). All xml files are available for download at https://www.hiv.lanl.gov/repository/BEASTxmlFiles/.

PF automatically screens for APOBEC-mediated enrichment of G-to-A substitutions (11), and alignment positions with a G in the context of an APOBEC motif were removed either (i) when hypermutation was found to be significant across a sample or within a sequence or (ii) when removal of the APOBEC positions improved the initial Poisson fit (5). For these samples, we used the APOBEC-removed alignment for the BEAST runs as well for a fair comparison.

When multiple TFs were detected, mutation frequency count distributions were obtained from each lineage separately, and the time since infection was calculated by fitting a Poisson distribution to the pooled mutational distances (14) (see PF help at https://www.hiv.lanl.gov/content/sequence/POISSON_FITTER/pfitter_help.html). Since distinct TFs were artificially designed to differ at one or two codons at most, we also analyzed these samples a second time after removing the codons at which the lineages differed to see if this alternative approach improved either the PF estimates or the BEAST estimates. No significant change was noted in the PF results when we removed the relevant sites, but, as expected, removing these sites improved the BEAST estimates. However, even using this approach, overall estimates and 95% CIs across all samples obtained through PF remained statistically significantly more accurate than the ones obtained from BEAST; the differences in the number of estimated days since infection and the known inoculation day were still statistically significantly lower for PF than for BEAST (*P* = 0.017 by paired Wilcoxon test), and the PF 95% CIs were still statistically significantly narrower than the BEAST CIs (*P* = 1.2 × 10^−7^ by paired Wilcoxon test).

Additional BEAST runs were implemented with the same parameters as those described above but with a coalescent Bayesian skyline prior, but this setting yielded worse estimates (data not shown). Finally, an additional run was implemented using data from five animals (RM6434, RM6442, RM6446, RM6454, and RM43335), all sampled at 28 days and with the same inoculum history, in order to attempt to estimate the molecular clock rate and then use such a rate to infer the time since infection. A molecular clock rate of 1.8 × 10^−5^ mutations per day yielded good time estimates; however, this was obtained through the use of BEAST after removal of all APOBEC positions and of the sites where the distinct infecting TFs differed. Again, we note that in an ordinary setting, where the inoculum and infection times are typically not known, BEAST does not provide ways to screen for these biases. Thus, the use of PF to identify these artifacts and to deal with them prior to using either PF or BEAST to estimate the time of infection from a sample, was advantageous.

### Data availability.

SGA sequences generated for each animal were deposited in GenBank under accession numbers MN467402 to MN472740 (https://www.ncbi.nlm.nih.gov/Genbank/). All xml files used for the BEAST runs, as well as the fasta files used as input, are available for download at https://www.hiv.lanl.gov/repository/BEASTxmlFiles. Poisson Fitter is freely available on the Web at https://www.hiv.lanl.gov/content/sequence/POISSON_FITTER/pfitter.html.
